# Magnetic Resonance Microscopy (MRM) of Single Mammalian Myofibers and Myonuclei

**DOI:** 10.1038/srep39496

**Published:** 2017-01-03

**Authors:** Choong H. Lee, Niclas Bengtsson, Stephen M. Chrzanowski, Jeremy J. Flint, Glenn A. Walter, Stephen J. Blackband

**Affiliations:** 1 Center for Biomedical Imaging, Department of Radiology, New York University School of Medicine, New York, NY, 10012, USA; 2 Chamberlain Laboratory, University of Washington, Department of Neurology, Seattle, Washington, 98195, USA; 3 Department of Physiology and Functional Genomics, University of Florida College of Medicine, Gainesville, FL, 32610-0274, USA; 4 Department of Neuroscience, University of Florida College of Medicine, Gainesville, FL, 32611, USA; 5 McKnight Brain Institute, University of Florida, Gainesville, FL, 32611, USA; 6 National High Magnetic Field Laboratory, Tallahassee, FL, 32310, USA; 7 Department of Physiology and Functional Genomics, University of Florida College of Medicine, Gainesville, FL, 32610-0274, USA.

## Abstract

Recently, the first magnetic resonance microscopy (MRM) images at the cellular level in isolated mammalian brain tissues were obtained using microsurface coils. These methods can elucidate the cellular origins of MR signals and describe how these signals change over the course of disease progression and therapy. In this work, we explore the capability of these microimaging techniques to visualize mouse muscle fibers and their nuclei. Isolated myofibers expressing *lacZ* were imaged with and without a stain for β-galactosidase activity (S-Gal + ferric ammonium citrate) that produces both optical and MR contrast. We found that MRM can be used to image single myofibers with 6-μm resolution. The ability to image single myofibers will serve as a valuable tool to study MR properties attributed to healthy and myopathic cells. The ability to image nuclei tagged with MR/Optical gene markers may also find wide use in cell lineage MRI studies.

The ability to monitor the onset and progression of disease, as well as evaluate therapeutic efficacy at the cellular level in a non-invasive and non-destructive manner, contributes to the understanding of disease etiology and provides more information for clinicians. To date, the most prevailing methodology to assess cellular status is through fluorescent microscopy techniques^1^,^2^,^3^,^4^. Through the use of reporter genes cells have been imaged at the whole body and tissue level using positron emission tomography, fluorescent mediated tomography, and luminescence where traditional marker genes and fluorescent protein are commonly implemented for detection on the cellular level. However, light-based technologies possess inherent underlying limitations, primarily due to photon attenuation in the visible wavelength range, limiting penetration depth within biological samples.

As a complementary methodology, magnetic resonance imaging (MRI), a versatile, non-ionizing, and non-invasive diagnostic imaging modality, has been employed to study the distribution of water molecules in the muscle cells of barnacles^5^,^6^, frogs^7^,^8^, rats^9^,^10^,^11^, and humans^12^. Due to recent advances in hardware and software which have improved spatial resolution in MRI, magnetic resonance microscopy (MRM) has transitioned from a technique used to image single, large cells, such as frog eggs^13^ and neurons of gastropod mollusks^14^,^15^,^16^,^17^,^18^, to much smaller cells such as mammalian α-motor neurons in rats^19^, pigs and humans^20^. Furthermore, it has recently proven its capability of visualizing the 3D brain connectivity in the fly brain based on endogenous contrast, which is the first map from the whole animal head^21^. By utilizing MR contrast mechanisms such as NMR relaxation and diffusion, MRM can take advantage of endogenous subcellular contrast, complementing other microscopy techniques.

*In vivo* cellular tracking is achieved through a combination of novel exogenous markers and state-of-the-art imaging techniques. Specifically, iron and gadolinium based contrast agents have been utilized to enhance spin-spin (T_2_) and spin-lattice (T_1_) relaxivity, which contain iron and gadolinium based products, respectively. Cellular iron-based contrast agents have been used to monitor gene regulation by upregulating and/or overexpressing proteins that bind cellular iron and result in changes in the MR signal through locally induced magnetic field inhomogeneities. These inhomogeneities in turn lead to an amplification of negative contrast and detectability by the reduction of T_2_ or T_2*_ relaxation times^22^,^23^. This approach can also be used to track cells exogenously labeled with superparamagnetic particles^24^,^25^. Due to the increased induced magnetic field inhomogeneities this has been used to detect single cells *in vitro*^26^ and *in vivo*^27^. It can also be used to assess transgene expression using the beta-galactosidase reporter system in translational research studies^22^,^28^,^29^,^30^ as well as cancer clinical trials^31^,^32^. Other commonly employed cellular contrast agents include gadolinium-loaded nanoparticles, which increase T_1_ relaxation rates of neighboring water molecules^33^,^34^. However, both contrast agents possess shortcomings in demarcating spatial characteristics at the cellular level due to the low specificity and spatial resolution of MRI. Such limitations warrant further investigation into these and alternative contrast agents including confirmation of their ability to accurately represent spatial relationships in the microscopic domain by correlative histological analysis^35^.

In this study, we employed the *lacZ* gene reporter system under control of the mouse myosin light chain 3 F promoter/enhancer element^36^ to detect β-galactosidase (β-gal) activity in cell nuclei^22^,^37^. Intact, single extensor digitorum longus (EDL) myofibers were harvested from wild-type control (C57/BL6) mice or transgenic mice expressing muscle-specific nuclear *lacZ*. Isolated fibers were labeled with S-Gal (3,4-Cyclohexenoesculetin β-D-galactopyranoside; Sigma-Aldrich, St. Louis, MO) and Ferric Ammonium Citrate (FAC). S-Gal is a commercially available histological stain for *lacZ*, while FAC allows for *in vitro* detection of β-gal myonuclei through negative contrast on T_2*_-weighted MRI scans^38^,^39^. The stained fibers from our transgenic strain were compared with unlabeled control fibers with S-Gal and FAC. To optimize our findings, fibers were doped with different concentrations of S-Gal and FAC to determine ideal staining conditions for the myofiber nuclei. Resolution and signal-to-noise ratio (SNR) were adequate for delineation of myonuclei demonstrating the complementary role of MRM to other methods of optical microscopy. Such high signal and resolution characteristics were achieved by using state-of-the-art radiofrequency (RF) micro coils^40^ in conjunction with strong and fast switching gradient coils^41^.

A representative schema of the mechanism of the experimental model is presented in Fig. 1. Because of the mouse myosin light chain (MLC) 3F promotor/enhancer element, expression of *nLacZ* (and subsequent translation of β-galactosidase (β-gal)) is specific to muscle. Briefly, S-Gal and FAC form Fe^3+^ in the presence of β-galactosidase (β-gal) resulting in visible black precipitate^38^. Depending on the length of incubation and concentrations of substrates, optical and MR contrast can be optimized to enhance contrast.

## Results

Using MRM, we were able to image single muscle fibers directly employing only native tissue contrast (Fig. 2) with a 3D fast low-angled shot (FLASH) sequence. In this experiment, an isotropic resolution of 6 μm and a SNR of 20 were achieved. Alternatively, using a 3D spin echo sequence, the spatial resolution was 8 × 8 × 31 μm and the SNR was 14. MRM using 3D FLASH and spin echo sequences demonstrates native contrast generated by the muscle fibers at 6 μm isotropic resolution (Fig. 2a) and 8 μm in-plane resolution (Fig. 2b). A direct comparison between morphological images of isolated muscle fibers using MRM (Fig. 2a and b) and differential interference contrast (DIC) microscopy (Fig. 2c) demonstrated the normal birefringence due to sarcomeric striations consisting of dark A-band and bright I-band in an interdigitated morphology (inset of Fig. 2c), and indicated that the fiber structures were intact. The relative scale of the muscle fiber and MR RF micro surface coil are shown in Fig. 2(d), as observed by light microscopy.

In order to confirm the identity of anatomical structures in our MR images, a genetic label capable of both histological and MRI contrast was used. Incubation with S-Gal/FAC allowed us to visualize muscle cells that were expressing *lacZ* by forming an opaque precipitate in the cytoplasm or nucleus. This ferric iron (Fe^3+^) precipitate was visible in both 3D FLASH microimages (Fig. 3a and b) and light microscopy (Fig. 3c and d). Accumulated iron content along the individual fibers contrasted with neighboring regions due to heterogeneous uptake. Without uniformly controlled targeting or binding of the iron-containing S-Gal, the resultant T_2_ (or T_2*_) contrast has been amplified mostly around the punctate areas along the fiber wall (Fig. 3a and b). Four myofibers were selected under the light microscope for possessing maximal differential phase response before being embedded into an agarose gel for MR imaging (Fig. 3c).

MRM of single myofibers using 3D FLASH enabled visualization of myonuclei at the shortest echo time used (TE = 1.5 ms) and detection of individual nuclei with 6 μm isotropic resolution in consecutive slices with a total thickness of 18 μm (Fig. 4a–c, red arrowheads). The spatial distribution of nuclei was visualized using 3D segmentation (Fig. 4d). We could detect precipitate in the FAC/S-Gal-doped myofiber after only 15 minutes of staining (Fig. 4e) by light microscopy. The selective distribution and shape of localized contrast inhomogeneities and hypointense signals around/at the nuclei in MRM (Fig. 4a–c) matched the pattern of distributed nuclei in differential interference microscopic images of myofibers expressing *lacZ* stained with FAC/S-Gal (Fig. 4e).

A group of *lacZ*-expressing myonuclei in myofibers that were stained for 15 minutes with FAC/S-Gal was visualized using bright-field microscopy (Fig. 5a). By employing the bigger diameter coil, i.e. 500 μm, for the wider coverage, stained nuclei demonstrated hypointense contrast in T_2_ (or T_2*_) images along the individual fibers in 3D FLASH MRM with 8 μm resolution (Fig. 5b). The through-plane arrangement of the coil was differentiated in such a way that the closer portions of the fiber was tilted vertically in a diagonal direction to the coil surface along with the remaining fibers including the clear demarcation of the fiber placed horizontally in the middle of the field of view (FOV). Spin echo images (in-plane resolution = 8 μm) also detected iron labeling in the projected image of entire geometry and arrangement of the myofibers, i.e., X-shaped geometrical configuration of intersection of myofibers at the center of the FOV for the landmark, but the spatial localization of individual nuclei was not possible because of the thicker through-plane resolution employed (160 μm) (Fig. 5c and d). By increasing the echo time from 5.8 (Fig. 5c) to 13.5 ms (Fig. 5d), the hypointense MRM signals emanating from S-Gal labeled myofibers resulted in noticeably amplified contrast.

Temporal and regional changes in iron-deposition on single myofibers were compared in Fig. 6. Figure 6(a) presents the myofiber after doping with S-Gal/FAC for 5 minutes. Expectedly, the amount of S-Gal/FAC deposition on the myonuclei was comparatively less than that of the myofibers which were incubated for 15 minutes (Fig. 5a). After an incubation time of 45 minutes, the myofiber became saturated with iron-containing S-Gal/FAC resulting in obfuscation of the labeled nuclei (Fig. 6b). The myofiber became entirely iron-saturated and opaque after an incubation time of 60 minutes (Fig. 6c).

## Discussion

**Figure 1:**
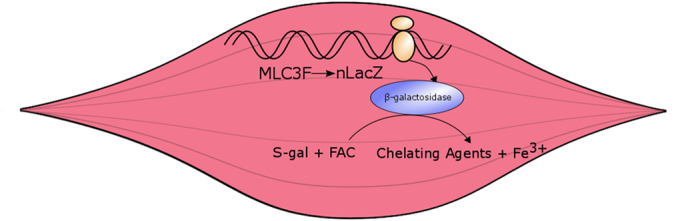
Schematic representation of MR gene reporter. Illustrative description of MLC 3F promotor/enhancer element to detect B-gal activity in myofibers. Generation of β-galactosidase in the presence of S-Gal/FAC causes precipitation of ferric iron (Fe^3+^): the presence of which allows for enhancement of both optical and MR contrast.

To the best of our knowledge, this study is the first to report visualization of individual mammalian muscle fibers and nuclei using MRM methods. Muscle nuclei were highlighted by quickly decaying T_2_ signals using *lacZ* as an anatomically targeted gene reporter following incubation with S-Gal/FAC^38^,^39^ (Fig. 1).

**Figure 2:**
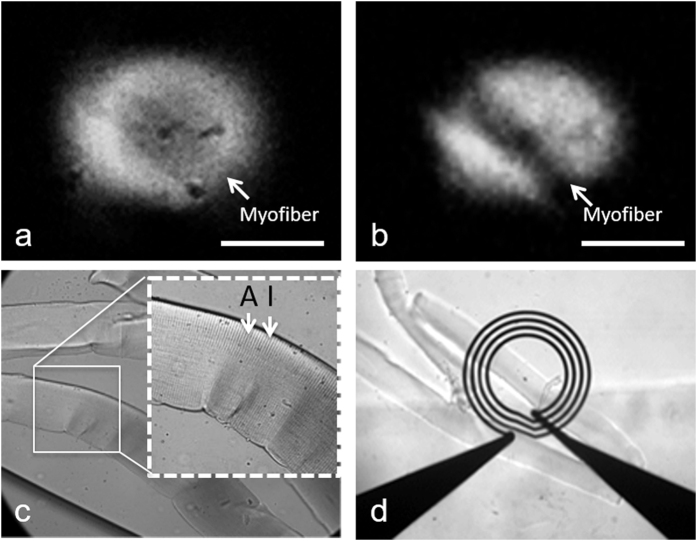
MRM, differential interference contrast microscopy (DIC), and bright-field microscopy of control muscle fibers. (**a**,**b**) Three-dimensional MRM of unstained, control muscle fibers (6 μm isotropic in a, 8 μm in-plane resolution in **b**,**c**) comparative DIC microscopy of C57/BL6 muscle fibers embedded in agarose block highlighting representative birefringent A- and I- band of the sarcolemma in the inset, and (**d**) positioning of the coil under bright-field microscopy are represented. MRM scan parameters: (**a**) 3D fast low-angle shot (FLASH) sequence with TE/TR = 2.8/500 ms, resolution = 6 μm^3^, FOV = 0.8 mm × 0.8 mm × 0.4 mm, matrix = 128 × 128 × 64, bandwidth = 100 kHz, read and phase gradient amplitudes = 23760 and 25080 mT/m, NEX = 26, acquisition time = 29 hours 34 minutes and (b) 3D Spin Echo (SE) sequence with TE/TR = 30/1000 ms, resolution = 8 × 8 × 31 μm^3^, FOV = 0.8 mm × 0.8 mm × 0.5 mm, matrix = 100 × 100 × 16, bandwidth = 50 kHz, read and phase gradient amplitudes = 1182 and 1094 mT/m, NEX = 8, acquisition time = 7 hours 6 minutes, temperature = 23 °C. Scale bar: 200 μm.

**Figure 3:**
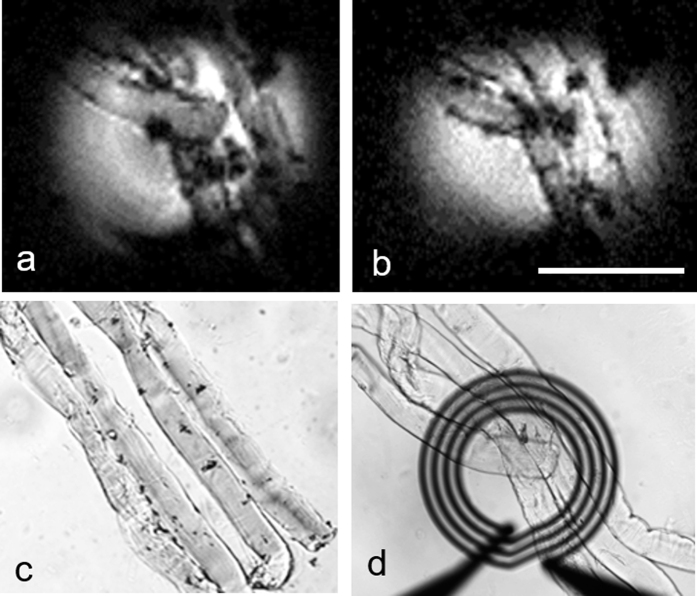
MRM, differential interference contrast (DIC), bright-field microscopy of control muscle fibers incubated with S-Gal/FAC. (**a**,**b**) Two consecutive slices from three-dimensional FLASH MRM (6 μm isotropic) of control muscle fibers after being incubated with S-Gal/FAC, (**c**) comparative DIC microscopy of C57/BL6 muscle fibers embedded in agarose block, (**d**) positioning on the coil under bright-field microscopy are shown. MRM scan parameters are as follows: 3D FLASH sequence with TE/TR = 2.8/500 ms, resolution = 6 μm^3^, temperature = 23 °C, FOV = 0.8 mm × 0.8 mm × 0.4 mm, matrix = 128 × 128 × 64, bandwidth = 100 kHz, read and phase gradient amplitudes = 23760 and 25080 mT/m, NEX = 26, acquisition time = 29 hours 34 minutes, Scale bar: 200 μm.

**Figure 4:**
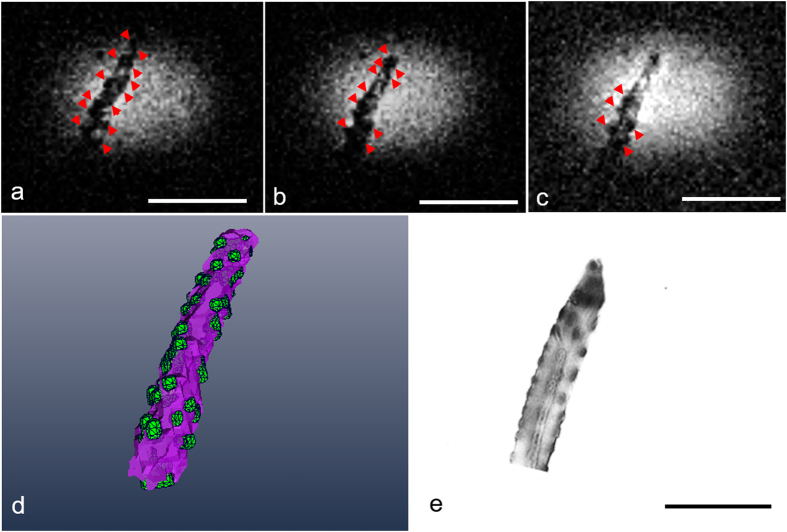
MRM, 3D visualization, and differential interference contrast microscopy (DIC) of S-Gal-labeled *lacZ*-expressing single muscle fiber. (**a**–**c**) Three consecutive slices from three-dimensional FLASH MRM of S-Gal-labeled *lacZ*-expressing muscle fibers at 6 μm each isotropic resolution out of 18 μm total thickness. (**d**) 3D reconstruction of a single muscle fiber consisting of S-Gal-labeled *lacZ*-expressing myonuclei (green) and intracellular structures (purple). (**e**) Comparative DIC microscopy of S-Gal-labeled *lacZ*-expressing myofiber embedded in agarose. MRM scan parameters: 3D FLASH sequence with TE/TR = 1.5/500 ms, resolution = 6 μm^3^, temperature =  23 °C, FOV = 0.8 mm × 0.8 mm × 0.4 mm, matrix = 128 × 128 × 64, bandwidth = 100 kHz, read and phase gradient amplitudes = 23760 and 25080 mT/m, NEX = 26, acquisition time = 29 hours 34 minutes, Scale bar: 200 μm.

**Figure 5:**
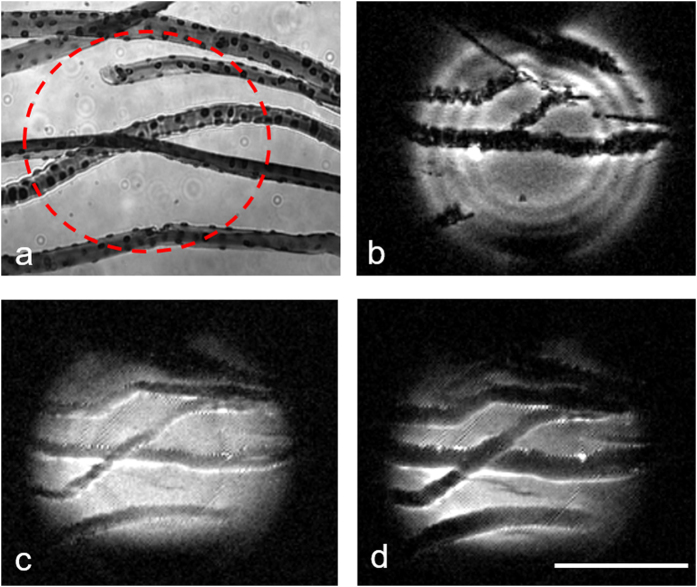
Bright-field microscopy and MRM of a group of S-Gal-labeled lacZ-expressing muscle fibers. (**a**) Bright-field microscopy of myofibers labeled with S-Gal together with *lacZ*-expressing myonuclei (black dots) embedded in agarose block (red circle [dashed line] indicates the sample region visualized in MRM), (**b**) 3D FLASH MRM of the same tissue sample at 8 μm isotropic resolution. MRM scan parameters: 3D FLASH sequence with TE/TR = 3.5/300 ms, temperature = 23 °C, FOV = 2 mm × 2 mm × 0.5 mm, matrix = 256 × 256 × 64, NEX = 14, acquisition time = 19 hours 6 minutes, (**c**) lower resolution of 2D T_2_-weighted spin echo images are presented. MRM scan parameters: 2D MSME sequence with TE/TR = 5.8/2000 ms, resolution = 8 × 8 × 160 μm^3^, temperature = 23 °C, FOV = 2 mm × 2 mm, matrix = 256 × 256, NEX = 30, acquisition time = 4 hours 16 minutes, (**d**) the same slice at an increased TE of 13.5 ms. Scale bar: 500 μm.

**Figure 6:**
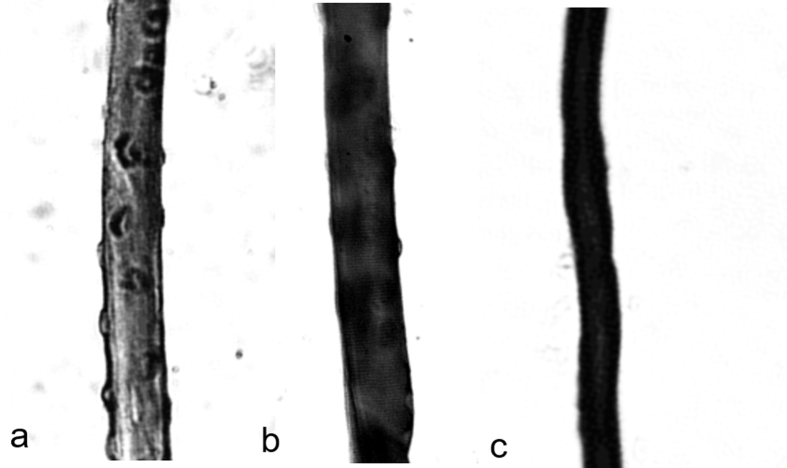
Bright-field microscopy of temporal differences in labeling *lacZ*-expressing muscle fibers with S-Gal. Bright-field microscopy of *lacZ*-expressing myofibers stained with S-Gal/FAC for (**a**) 5 minutes, (**b**) 45 minutes and (**c**) 60 minutes. The level of fiber opacity increases—alongside a concurrent decrease in nuclear contrast—with increasing incubation time.

To test the specificity of *lacZ* as a genetic reporter and the role of β-galactosidase activity, we incubated C57/BL6 myofibers with S-Gal/FAC (Fig. 4). In the negative control fibers containing no S-Gal/FAC staining, there was no contrast in 3D FLASH (Fig. 2a) or 3D spin echo MRM (Fig. 2b). By contrast, in the stained fibers, the areas where S-Gal was taken up demonstrated T_2_ (or T_2*_) contrast enhancement, primarily in punctate areas surrounding the fiber (Fig. 3a and b). However, because of the low efficiency of myonuclei targeting as well as the non-uniform distribution of iron-containing S-Gal, these techniques alone could not demarcate the precise locations of myonuclei. The occurrence of excessive background staining of iron is in strong agreement with our previous study^39^. Building upon the reported detectability of T_2_ (or T_2*_) signal after S-Gal/FAC incubation of *lacZ*-expressing myofibers, this study is the first to address the optimization of incubation duration (15 minutes) and spatially appropriate resolution for visualizing myonuclei with MRM (6 μm) (Fig. 4).

Subcellular resolution was achieved using dedicated gradient coils with 66 T/m strength and 1.1 T/m/A slew rate^41^. These hardware characteristics allowed us to overcome bandwidth and diffusion-based resolution limitations, as discussed previously^19^,^41^,^42^. Because of the linear relationship between iron concentration and the external magnetic field, the required minimum concentration of ferritin and exposure time to generate T_2_ (or T_2*_)-based contrast could be reduced 10-fold. Such concentrations of iron are smaller than those previously demonstrated^43^. This may be an additional and relevant benefit to use T_2_ -based contrast agents in high field MRM rather than T_1_-based agents, as T_1_-based agents’ contrast becomes smaller in proportion to the strength of the external magnetic field^44^.

Investigation into the microstructural underpinnings of subcellular compartments using endogenous MR contrast has been reported in nervous tissue systems including isolated *aplysia* neurons (300–500 μm)^14^,^15^,^16^,^17^,^18^,^45^,^46^, rat^19^ and human α-motor neurons^20^, and xenopus gamete cells^13^,^47^,^48^,^49^,^50^,^51^. The subcellular regions of the *Aplysia* L7 neuron visualized using native MR contrast were recently identified through correlative histological analysis^18^. By contrast, there have not been similar studies to investigate MR signal characteristics at the subcellular level in skeletal muscle in large part due to the low sensitivity and specificity in MRI. In this study, by highlighting the efficient contrasting effect by enlarged surface area of exogenous contrast agents in isolated individual muscle cells, the microstructural origin of contrast enhanced subcellular structures such as myonuclei was identified clear enough to differentiate the linear relationship between exposure time to FAC, the empirical threshold temporal information, i.e., 15 min exposure time, and contrast enhancement for the identification of the subcellular targeting for potential therapeutic applications with fine tunability. With the technical improvement of sensitivity and resolving power of MRM, these results warrant further investigation on diagnostic utility of (sub) cellular profiles and possible role in cellular tracking based on endogenous contrast. With the evolving technical improvements to sensitivity and resolving power offered by MRM, these results suggest further investigation into the potential diagnostic utility of sub-cellular, tissue-specific labeling methods is warranted.

The *lacZ* gene reporter system presented in this study may further expand the ability of MRM to non-invasively and longitudinally monitor transgene expression. Specifically, this technology may be useful for pre-labeling of rapidly proliferating stem cells *in vitro*, in order to follow their migration to their final destination *in vivo*. Furthermore, with natural reporter systems, direct *in vivo* mapping may be possible based on gene reporter expression in transgenic animal models. Such tools could lead to a better understanding of the cellular mechanisms responsible for a multitude of human pathologies including muscular dystrophies and cancers.

Regarding translational issues, we do not anticipate these spatial resolutions to be possible in the foreseeable future in human studies due to the intrinsic hardware requirements. Rather, these works will enable us to understand the cellular origins of the signal changes seen in clinical MRI (or animal studies) using these gene markers. In this study, we chose to demonstrate the use of a generic gene marker under the control of a very specific gene muscle promoter. In our experience the extrapolation to *in vivo* studies is not limited by the promoter but by the substrate availability of S-gal and the iron. Whereas we have shown in the past this reporter system can be used in mice^39^ and others have demonstrated its use in a *in vivo* tumor model it relied on highly tissue permeability *in vivo*^30^. Human represents further challenges due to the need to introduce a marker gene without clinical benefit, the off-label use of iron supplementation, and the approval to use S-gal in humans. For these reasons, this MR reporter is best utilized as demonstrated in this study as a MR histological stain.

### Conclusions and Future Work

In summary, this study has demonstrated an *in vitro* method for using MRM as a tool to study myonuclei of individual muscle fibers. Similar methodology may offer novel means to non-invasively track cells *in vivo*. This type of highly efficient cellular labeling may allow for MR-based cell tracking to serving a vital role in the study and monitoring of therapeutic interventions for a wide variety of human diseases. As an alternative to invasive biopsy, high resolution MRM is a non-destructive, non-ionizing technology that can visualize tissue dynamics using unique contrast. Such methods could provide a more useful, tissue-specific protocol for cellular tracking and potentially aid in the early detection of pathology using molecular imaging techniques. In conclusion, the findings from this study demonstrate that cellular MRM in mammalian muscle fibers is feasible, and that—when combined with histological labeling techniques–MRM allows for detection of subcellular components in mammalian tissue: myonuclei. Future studies will focus on development of protocols from molecular biology into MRI techniques for diagnostic clinical medicine.

## Methods

### Muscle Fiber Preparation

All animal procedures were conducted in accordance with guidelines in the National Academies of Sciences’ Guide for the Care and Use of Laboratory Animals and were approved by the University of Washington IACUC. Single myofibers were isolated from dissected extensor digitorum longus (EDL) muscles of wild type (C57/BL6) and transgenic MLC3FnLacZ mice. Intact EDL muscles were digested with DMEM containing 4 mg/mL Collagenase type 1 (Sigma-Aldrich, St. Louis, MO, USA) for 1 hour at 37 °C, followed by manual trituration using fire polished glass Pasteur pipettes to release individual fibers (as described in Keire *et al*.)^52^. Isolated fibers were fixed for 5 minutes in 2% formaldehyde and stored in phosphate-buffered saline (PBS) at 4 °C until staining and MRM. Myofibers were individually separated in a petri dish of culture medium. Then, a 1:1 molar ratio of S-Gal: FAC solution was mixed with 1% PBS and added to the culture dish containing fibers. These were allowed to incubate in a 37 °C water bath for up to 1 hour.

### Intracellular S-Gal Staining and Specificity of Myofibers

After heating the water bath to 37 °C, isolated myofibers were incubated in the stock solution of 1 mg/mL S-Gal (Sigma-Aldrich, St. Louis, MO) with 0.5 mg/mL ferric ammonium citrate (FAC) (Sigma-Aldrich, St. Louis, MO) dissolved in PBS (Fig. 1). After incubation times of 5, 15, 45, or 60 mins, myofibers were copiously rinsed with PBS and transferred with fire-polished Pasteur pipettes (803 A, Wilmad-LabGlasses) to fresh PBS.

### Sample Positioning, MRM, and 3D Visualization

All MRM was carried out on a 600 MHz (14.1 T) vertical-bore magnet (Oxford Instruments) interfaced to a Bruker Biospin console. Imaging gradient strengths up to 66 T/m were provided by a newly designed and fast-switching (1.1 T/m/A) planar gradient system (Bruker Biospin, Z110828, B6406). Initially, myofibers were embedded in low-melting point agarose (22-110-617, Fisher) and positioned by hand with the aid of a dissecting scope (Zeiss, OPMI 1-FC). Next, an agarose block containing embedded samples was placed directly on the RF microcoil. Lastly, the tissue well was sealed using PCR film (ABgene, AB-0558) to prevent leakage and ensure the physical stability of samples. Imaging protocols were repeated for each stain concentration and incubation time tested (n = 3).

In MRM experiments targeting myonuclei, micro surface-coils of 200 μm (inner diameter) (Bruker Biospin, B6371) and 500 μm (inner diameter) (Bruker Biospin, B6370) were utilized. Three-dimensional FLASH datasets containing the nucleus and neighboring intracellular regions of single muscle fibers were segmented, analyzed, and reconstructed using 3D image analysis software (Amira 5.4.0; Visage Imaging) in order to visualize subcellular structures from multiple angles.

## Additional Information

**How to cite this article**: Lee, C. H. *et al*. Magnetic Resonance Microscopy (MRM) of Single Mammalian Myofibers and Myonuclei. *Sci. Rep.* **7**, 39496; doi: 10.1038/srep39496 (2017).

**Publisher's note:** Springer Nature remains neutral with regard to jurisdictional claims in published maps and institutional affiliations.
